# Rapid antigenic test (TRA) versus RT-PCR: Experience of CHU IBN ROCHD Casablanca

**DOI:** 10.1016/j.amsu.2022.103908

**Published:** 2022-06-05

**Authors:** O. Elfadel, F. Zannane, M.Soussi Abdallaoui

**Affiliations:** a Bacteriology-virology Laboratory, CHU IBN ROCHD, Casablanca, Morocco; b Faculty of Medicine and Pharmacy, Hassan II University, Casablanca, Morocco

## Abstract

• Ag-RDTs have a good specificity varying from 93.2 to 100% depending on the kits in comparison with RT-PCR.
• In our study, the specificity of the BIOSENSOR® test was 100% and the sensitivity was 47.17%.
• This drop of the specificity is explained by the decrease of the viral load with the progression of the infection.

## 1 Introductionintroduction

SARS-CoV-2 is an enveloped virus that belongs to the genus Betacoronavirus (subgenus Sarbecovirus) of the family Coronaviridae [1]. Its genome is composed of a single-stranded ribonucleic acid (RNA). The virus is characterized by an RNA correcting mechanism that keeps the possible mutation rate at a relatively low level.

The genome encodes non-structural proteins, among which, some are essential for the establishment of the replicase-transcriptase complex. The four main structural proteins are Spike (S), the envelope protein (E), the membrane protein (M), the nucleocapsid protein(N), and the putative accessory proteins [2].

The confirmation of acute SARS-CoV-2 infections relies on the detection of viral sequences using nucleic acid amplification assays (NAAT), mainly represented by Real time polymerase chain reaction (reverse transcription followed by gene amplification). The targets of these techniques involve regions located on the E, RdRP, N and S genes [3].

The detection of SARS-CoV-2 in the upper respiratory tract may be attainable 1–3 days before clinical symptoms, however the concentration of the virus remains the highest at symptom onset and abates gradually [5].

After the incubation period, the average delay to the clinical manifestation is 5–6 days, with extremes ranging from 1 to 14 days after exposure [4].

When a Covid-19 infection is suspected, appropriate samples must be collected to obtain a biological diagnosis, in order to optimize the clinical management of the patients and also to control the spread of infection, but the complexity of sample acquisition, analyses and results interpretation requires qualified operator.

These limitations have brought about scientists to seek more rapid and precise diagnostic methods directed towards expanding testing capacity and controlling the global pandemic situation. A large number of diagnostic tests have been developed and marketed in a very short time, from gold standard, RT-PCR, to antigenic and serological tests [11].

Antigenic tests are unitary tests, shaped in easy-to-handle cassettes that can be carried out by medical biology laboratories. They grant the detection of one of the SARS-CoV-2 virus proteins, generally the nucleocapsid protein, and thus allows, like the detection of the viral RNA by the reference technique (RT-PCR), to make a positive diagnosis during the early phase which accelerates and facilitates the diagnostic process reducing then the risk of viral transmission.

The aim of the present study is to explore the sensitivity (true positive rate) and specificity (true negative rate) of the antigen test compared to RT-PCR in order to be able to compare the performance of the test to different studies conducted in the same period involving the development and potential usefulness of the COVID-19 Ag Diagnostic Assay in a Pandemic Context like Mertens P study [15], Dr Slim FOURATI testing the performance of six antigen rapid tests [10,14],and the WHO recommendations on Antigen-detection in the diagnosis of SARS-CoV-2 infection [7].

This work has been reported with respect to the PROCESS 2020 criteria [16].

## 2 Material and methods

### 2.1 Methods

This is a prospective comparative study conducted at the Bacteriology-Virology Laboratory of the Ibn Rochd University Hospital in Casablanca (Morocco) over a period of 3 months from 10 January 2021 to 20 March 2021, including patients treated in our laboratory for whom nasopharyngeal samples were for diagnosis of COVID-19.

## 3 Material

• **Antigenic tests:**

All samples were tested systematically for antigens using the STANDARD COVID 19 Ag BIOSENSOR® kit which is a unitary test based on an immunochromatographic technique.

The kit contains: A test device, an Extraction buffer tube, a Nozzle cap, a sterile swab and Instructions for use.

This test has two pre-coated lines, “C” Control line, “T” Test line on the surface of the nitrocellulose membrane. Both the control line and test line in the result window are not visible before applying any specimens.

Mouse monoclonal anti-SARS-CoV-2 antibody is coated on the test line region and mouse monoclonal anti-Chicken IgY antibody is coated on the control line region.

Mouse monoclonal anti-SARS-CoV-2 antibody conjugated with color particles are used as detectors for SARS-CoV-2 antigen device.

During the test, SARS-CoV-2 antigen in the specimen interacts with monoclonal anti-SARS-CoV-2 antibody conjugated with color particles making antigen-antibody color particle complex.

This complex migrates on the membrane via capillary action until the test line, where it will be captured by the mouse monoclonal anti-SARS-CoV-2 antibody.

A colored test line would be visible in the result window if SARS-CoV-2 antigens are present in the specimen.

The control line is used for procedural control, and should always appear if the test procedure is performed properly and the test reagents of the control line are working.

This test also permits rapid results to be obtained: 15–20 min.

The test was performed according to the manufacturer's recommendations; in short: we added 350 μl of the sample to the buffer extraction solution, then we applied 3 drops of extracted specimen to the specimen well of the test device. The result is to read after 15–30 min.

• **RT-PCR:**

The RNA extraction was carried out by a magnetic bead-based method provided by the Maxwell promega kit, then, the amplification was performed on the Quant Studio5 Thermofisher thermal cycler by the Moroccan kit Mascir® 2.0, the latter enables the detection of 2 genes, the RdRp gene and the one encoding the S protein.

The PCR results interpretation was made according to the number of genes detected and their Ct values:

• Cts >37 meant a negative RT-PCR
• The detection of 2 genes with Cts <35 confirmed the COVID-19 diagnosis.
• The detection of only 1 gene with a Ct < 34: Extraction and RT-PCR were performed again. When the same result was obtained the diagnosis of COVID-19 was confirmed.
• 35< Cts <37: Re-extraction and RT-PCR, when the same results, a control sample after a few days was requested.

## 4 Results

A total of 841 patients were admitted to the Ibn Rochd University Hospital during the study period for suspected SARS-CoV-2 infection.

The sex-Ratio was 0.73(357/484), with an average age of 35.

The diagnosis of COVID-19 was confirmed by RT-PCR in 159 patients, while for the other 682 patients, RT-PCR was negative.

## 5 BIOSENSOR® compared to RT-PCR (Table 1)

In 159 patients whose diagnosis was confirmed by RT-PCR; 75 came back positive by the antigen test, so a sensitivity of 47.17%, the remaining 84 were negative, which means 52.83%.

For all the patients having a negative PCR (N = 682), the search for viral antigen by the BIOSENSOR® kit was negative, with a specificity of 100%.

## 6 BIOSENSOR® test results correlated to RT-PCR Cts values (Table 2)

The comparison showed that the positivity of the antigen test is significantly correlated to the Ct values of the RT-PCR.

Indeed, from 27 patients with a positive RT-PCR and a Ct value ≤ 20, 25 had a positive antigen test, with a sensitivity of 92.59%.

for 38 patients with a positive RT-PCR and a Ct value: 20 <Cts≤25; 31 patients had a positive antigen tests, with a sensitivity of 81.58%.

For 39 patients with a positive RT-PCR and a CT value: 25 <Cts≤30, 16 patients returned positive with the BIOSENSOR® kit, with a sensitivity of 41.03%.

For 55 patients with a positive RT-PCR and a ct value: Ct > 30 (N = 55), only 3 patients had a positive antigen test, which means a sensitivity of 5.45% (Table 2).

## 7 BIOSENSOR® test results correlated to the moment of test performing (Table 3)

The positivity of the antigen test was also significantly related to the delay between symptoms onset and the performance of the RDT-Ag.

For the 53 patients with a delay ranging from 1 to 3 days, 33 had a positive antigen test (62.26%), for the 74 with a delay between 4 and 7 days, 31 turned positive with the BIOSENSOR test® (41.89%).

For the 32 remaining patients with a delay greater than 8 days, 11 patients only returned positive, which means 34.38%.

Therefore**,** the longer the delay, the more the sensitivity of BIOSENDOR TEST decreases.

## 8 Discussion

Considering the current situation of Covid-19 pandemic, the global strategy chosen to limit the spread of the virus aims to identify symptomatic patients or asymptomatic carriers of Sars-CoV-2, and to detect contagious cases to ensure their isolation as quickly as possible. For this purpose, efficient screening tools are needed. The gold standard biological test for Covid-19 is represented by RT-PCR. [6], results are normally available in less than 2 h, but most assays require laboratory facilities with robust infrastructure and highly trained staff, therefore, antigen rapid diagnostic tests (RDTs) were developed to accelerate and facilitate the diagnostic process.

Ag RDTs are generally qualitative methods performed on nasopharyngeal swab specimens. They use specific monoclonal antibodies for viral antigens that can be read using chromatographic particles.

Given the nature of the sample, they must be performed at the laboratory. The results are faster to obtain compared to RT-PCR (15–30 min) and do not require any equipment [8].

The robustness of an Ag-RDT is determined by its sensitivity and its specificity for the detection of a SARS-CoV-2 infection compared to nucleic acid amplification test, usually an RT-PCR [7].

Ag-RDTs have a good specificity [12], varying from 93.2 to 100% depending on the kits [10] in comparison with RT-PCR.Their sensitivity is however relatively low, and ranges between 55 and 62% according to a study of Slim Fourati interesting six rapid diagnostic tests for SARS-CoV-2 antigen detection and implications for practical use including our kit BIOSENSOR® and the results where almost identical, the specificity in Slim study of SARS-CoV-2 RDTs was generally high (398.5%). One assay had a lower specificity of 93.2%. The overall sensitivity of the 6 RDTs was variable, from 32.3% to 61.7% [14] than in our study, the specificity of the test was 100% and the sensitivity was 47.17%.

It should be noted that the efficiency of viral antigen detection depends directly on the viral load and therefore indirectly on the number of amplification cycles in quantitative RT-PCR than also the study of Vandenberg who calculated their performance based on a threshold cycle (Ct) below 22 and they concluded to found an overall sensitivity and specificity of 57.6 and 99.5% [12,15].

In fact, these tests are likelihood to show good sensitivity in patients with a high viral load (Ct values ≤ 25) [9], which is the case in the pre-symptomatic (1–3 days before symptoms onset) and early symptom phases (5–7 first days of symptoms) (7.

In our study, the positivity rate was 86.15% for samples with Cts ≤25 (Table 2), therefore our results go along with those of the literature.

**Table 1:** BIOSENSOR® antigen test results versus RT-PCR.

PCR	Ag RDT +		Ag RDT -	
	N	%	N	%
Positive n = 159	75	47.17%	84	52.83%
Négative n = 682	–	–	682	100%

**Table 2:** BIOSENSOR® test results correlated to RT-PCR Ct values.

Ct value ranges	Number of positive RT-PCRs	Number of positive BIOSENSOR® tests	BIOSENSOR® test: positivity rate
Ct < 20	27	25	92.59%
20≤Ct < 25	38	31	81.58%
25≤Ct < 30	39	16	41.03
Ct ≥ 30	55	3	5.45%

Moreover, we should recall that the positivity of these antigenic tests fluctuates according to the time between the symptoms' appearance and test performance: the shorter the period, the higher the sensitivity.

WHO recommends that SARS-CoV-2 Ag-RDTs that meet the minimum performance requirements of ≥80% sensitivity and ≥97% specificity compared to a NAAT reference assay can be used to diagnose SARS-CoV-2 in suspected COVID-19 cases. Clinical discretion considering epidemiological context, clinical history and presentation and available testing resources should determine if negative Ag-RDT results require confirmatory testing with NAAT or repeat testing with Ag-RDTs (within 48hrs) if NAAT is not readily available.

According to the literature recommendations, the positivity rate is 62.26% for patients with a delay between 1 and 3 days, whereas patients who consult beyond the 5th day after the start of symptoms have a high probability of obtaining false negative results with RDT-Ag [7].

In our series, the sensitivity decreases to 34.38% for patients with a delay greater than 7 days (Table 3). This drop of the specificity is explained by the decrease of the viral load with the progression of the infection.

**Table 3:** BIOSENSOR® test results correlated to the delay between symptom onset and test performing.

Delay between symptom onset and testing (days)	Positive PCRs	Positive BIOSENSOR® tests	BIOSENSOR® test: positivity rate
1–3	53	33	62.26%
4–7	74	31	41.89%
≥8	32	11	34.38%

## 9 Conclusion

Whilst Ag-RDTs are less sensitive than RT-PCR, they do have a good specificity. Indeed, the benefit of the quick results and the lower cost compared to RT PCR tests (100MAD VS 5OOMAD) would still overshadow the disadvantages of occasional false negatives.

All things considered Ag-RDTs, when used appropriately, are promising tools for scaling up screening [13], which justify their use in a pandemic context [6].

And the aim of our work was to compare the test performance of our Ag RDT test to the different other kits tests studied during the first wave of SARS-COV-2 in order to establish an easy, rapid and considerably reliable diagnostic method with the aim of enriching the scientific bibliography and sharing the experience of our department as well as in the future implications of establishing equally efficient diagnostic algorithms to capture the pandemic.

However, they cannot substitute RT-PCR particularly when negative in patients with high clinical suspicion, an RT-PCR must be then performed.

## Ethical approval

I declare on my honor that the ethical approval has been exempted by my establishment.

## Sources of funding

None.

## Author contribution

Elfadel Ouiame: Corresponding author writing the paper.

Zannane Fatima zahrae: writing the paper.

Soussi abdallaoui Maha: Study concept and correction of the paper.

## Trail Registry number

None.

## Guarantor

DR OUIAME ELFADEL.

## Annals of medicine and surgery

The following information is required for submission. Please note that failure to respond to these questions/statements will mean your submission will be returned. If you have nothing to declare in any of these categories then this should be stated.

## Consent

Written informed consent for publication of their clinical details and/or clinical images was obtained from the patient.

## Declaration of Competing interest

The authors declare having no conflicts of interest for this article.
